# Screening intrinsic capacity and its epidemiological characterization: a secondary analysis of the Mexican Health and Aging Study

**DOI:** 10.26633/RPSP.2021.121

**Published:** 2021-09-13

**Authors:** Luis Miguel Gutiérrez-Robledo, Rosa Estela García-Chanes, Mario Ulises Pérez-Zepeda

**Affiliations:** 1 Instituto Nacional de Geriatría Mexico City Mexico Instituto Nacional de Geriatría, Mexico City, Mexico

**Keywords:** Frailty, healthy aging, primary care, Mexico, Fragilidad, envejecimiento saludable, atención primaria de salud, México, Fragilidade, envelhecimento saudável, atenção primária à saúde, México

## Abstract

**Objective.:**

To describe the levels of intrinsic capacity and those factors related to its decline in Mexican older adults, using the Mexican Health and Aging Study.

**Methods.:**

This is a cross-sectional secondary analysis of the 2015 data of the Mexican Health and Aging Study, including adults aged 50 years and above. Selected questions were included to represent each domain of intrinsic capacity screening: cognition, depression, hearing, vision, anorexia, weight loss, and mobility. Sociodemographic characteristics, psychosocial factors, and health conditions were included to assess their association with intrinsic capacity. Further categories were established to assess not only individual characteristics but also different groupings. Along with descriptive statistics, multinomial regression models were performed.

**Results.:**

From a total of 12 459 adults aged 50 years and above, 54.7% were women and the average age was 71.2 years; 87.8% of the individuals had at least one intrinsic capacity domain affected, and mobility had the highest frequency (47.6%). All domains showed a trend of increasing with age and were higher among women. Self-rated health, chronic diseases, number of visits to a physician in the last year, and ≥2 affected activities of daily living were consistently associated with more intrinsic capacity domains affected.

**Conclusions.:**

Decreased levels of intrinsic capacity in Mexican older people are associated with less schooling, self-rated health, chronic diseases, visits to a physician, and activities of daily living.

## RESUMEN

**Objetivo.:**

Describir los niveles de capacidad intrínseca y los factores relacionados con su declive en las personas mayores en México, mediante un estudio sobre la salud y el envejecimiento en México.

**Métodos.:**

Se llevó a cabo un análisis secundario transversal de datos de un estudio sobre la salud y el envejecimiento en México realizado en el 2015 con adultos de 50 años o más. Se seleccionaron preguntas para cada ámbito de la exploración de la capacidad intrínseca: cognición, depresión, audición, visión, anorexia, pérdida de peso y movilidad. Se incluyeron las características sociodemográficas, los factores psicosociales y la condición de salud para evaluar su relación con la capacidad intrínseca. Se establecieron categorías adicionales para evaluar las características no solo individuales sino también en diferentes grupos. Junto con las estadísticas descriptivas, se llevaron a cabo modelos de regresión polinómicos.

**Resultados.:**

De un total de 12 459 adultos de 50 años o más, 54,7% eran mujeres y la edad promedio era 71,2 años; 87,8% de los participantes habían visto afectado al menos un ámbito de su capacidad intrínseca; la movilidad tuvo la frecuencia más alta (47,6%). Todos los ámbitos mostraron una tendencia al alza con la edad y se mostraron más elevados en las mujeres. La salud autoevaluada, las enfermedades crónicas, el número de consultas al médico el año anterior y ≥2 actividades cotidianas afectadas se asociaron sistemáticamente con una afectación de más ámbitos de la capacidad intrínseca.

**Conclusiones.:**

La reducción de los niveles de capacidad intrínseca de las personas mayores en México está asociada con una menor educación, una salud autoevaluada, enfermedades crónicas, consultas médicas y la actividad cotidiana.

## RESUMO

**Objetivo.:**

Descrever os níveis de capacidade intrínseca e os fatores relacionados ao seu declínio em idosos mexicanos, utilizando o Estudo Nacional de Saúde e Envelhecimento no México.

**Métodos.:**

Esta é uma análise secundária transversal dos dados de 2015 do Estudo Nacional de Saúde e Envelhecimento no México, que inclui adultos de 50 anos ou mais. Foram incluídas perguntas selecionadas para representar cada domínio de avaliação da capacidade intrínseca: cognição, depressão, audição, visão, anorexia, perda de peso e mobilidade. Características sociodemográficas, fatores psicossociais e condições de saúde foram incluídos para avaliar sua associação com a capacidade intrínseca. Outras categorias foram estabelecidas para avaliar não apenas as características individuais, mas também diferentes agrupamentos. Além de estatísticas descritivas, foram utilizados modelos de regressão multinomial.

**Resultados.:**

De um total de 12 459 adultos de 50 anos ou mais, 54,7% eram mulheres, e a idade média era de 71,2 anos; 87,8% dos indivíduos tinham pelo menos um domínio de capacidade intrínseca afetado, com mais frequência a mobilidade (47,6%). Todos os domínios tiveram tendência a aumentar com a idade e foram mais elevados entre as mulheres. A autopercepção de saúde, as doenças crônicas, o número de consultas médicas no ano anterior e ≥ 2 atividades da vida diária afetadas estavam consistentemente associados a um maior número de domínios de capacidade intrínseca afetados.

**Conclusões.:**

Níveis menores de capacidade intrínseca em idosos mexicanos estão associados a um menor nível de escolaridade, à autopercepção de saúde, a doenças crônicas, a consultas médicas e às atividades da vida diária.

Demographic aging is a well-established phenomenon around the globe, which has brought a number of challenges in health care for individuals in older age groups (1). One of the most urgent matters is that of providing adequate primary care for older adults, as almost every other older adult requires some level of attention in this stage of their lives, most likely in the form of primary care (2). To address this challenge, in 2015, the World Health Organization (WHO) and its partners put in place a strategy to boost the primary care of older adults to complement what the field of geriatrics already brings to this purpose (3). It also has the goal of increasing globally the number of older adults with so-called healthy aging. In order to achieve this ambitious plan, a number of actions are currently in place, including lobbying with different actors in the field (4). One of these actions is the creation of several novel concepts to increase the understanding of aging with a focus on primary care, the main area of the health system with which this age group will have contact. One of these concepts is that of intrinsic capacity (IC), defined by WHO as “a composite of all the physical and mental capacities of an individual” (5). Currently, the domains included in IC are: cognition, psychological, senses (vision/hearing), vitality, and mobility. Based on this capacity, a series of steps is proposed for evaluation of and intervention with older adults in order to defer the deterioration of their functional capacity (6).

IC has some similarities with frailty, the well-established condition that renders the older adult vulnerable to common stressors (7). Notably, IC has also been shown to be related to adverse outcomes in different settings (8, 9). As a broader concept including specific domains, IC has also been inversely related to allostatic load (10). Evidence on its appropriateness for insertion into the current practices of older adult care is still scarce; however, it has been launched as a core concept of the intervention planned by WHO, simplifying its assessment as a screening tool.

Integrated Care for Older People (ICOPE) was recently delineated in WHO guidelines, with the primary goal to prevent, slow, or reverse those declines that come with aging (6). As other initiatives on this matter have suggested, it has a multistage/multidisciplinary approach, including five general steps: screening, with further thorough assessment for those who screen positive, interventions according to the assessment, and follow-up with personalized goals (11). The starting point of these steps involves the implementation of a quick assessment of the domains of IC, which leads to a broader examination if any condition on the screening tool is abnormal. Some studies have described how this first step will look (12); however, since this strategy is planned to be used worldwide, there is a need for epidemiological information from around the world that characterizes the phenomenon in order to continue with the next steps in different regions and according to their needs.

Latin America, and especially Mexico (13), have an accelerated population aging phenomenon, with particular features that increase the complexity of care for the older adult (14). There is a continuously growing demand for sound information that will improve the health of the aging population, starting with its epidemiological characteristics. Consequently, the aim of this article is to describe the levels of IC and those factors related to its decline in Mexican older adults, using data from the Mexican Health and Aging Study (MHAS).

## MATERIALS AND METHODS

This is a cross-sectional analysis of MHAS data (Wave 2015). The specific objectives and procedures of the MHAS are fully described elsewhere (15, 16). In brief, MHAS aims at providing insight into the determinants of aging in the Mexican population. Starting in 2001, MHAS has five rounds of data collection (2001-baseline, 2003, 2012-sample refreshment, 2015, and 2018), with a follow-up round currently planned in 2021. Different domains are included in the questionnaires applied to adults aged 50 years and older, including social, economic, demographic, health-related, biomarkers, and mental health variables. Data are available, following registration, from the webpage http://www.mhasweb.org/.

### Variables

**Intrinsic capacity screening.** According to the proposal of the ICOPE guidelines (15), Step 1 comprises a series of tests used to detect a possible deterioration of each domain of IC: cognition, psychological, senses (vision and hearing), vitality, and mobility. Variables from the corresponding sets of MHAS questionnaires were selected to construct a screening tool (Table 1). For each of the domains, a score of 1 was assigned to a positive screening result. Scores were added to reach a single score, ranging from 0 to 6; further grouping was done according to this score into four groups considering the distribution of the variable: no affected domain (0), one affected domain, two or three, and four to six affected domains. The objective of categorizing in this way is to show the number of affected domains rather than summarize in a continuous variable.

Regarding cognition, verbal recall memory and orientation tests were used; having any of these wrong was considered a positive screening result. For the psychological domain, two questions from the MHAS symptoms of depression assessment (17) were used. Self-rated vision and hearing were used to assess these domains; screening was considered positive when the answer was fair, poor, or legally blind/deaf. Regarding vitality, weight loss and anorexia were evaluated as proxies for this domain. Finally, screening for mobility problems was positive if the individual responded “yes” to a question about having difficulty walking several blocks or climbing several flights of stairs without resting. It is important to mention that these mobility criteria may overestimate the decline in older ages because these activities require greater physical condition than walking one block or climbing one floor, which are also included. However, it was important to demonstrate, under these parameters, the situation of the Mexican population aged 50 and above.

Additional grouping was done following a preliminary analysis of the affected domains. Patterns were proposed for two or more affected domains to highlight the worst possible outcomes and their associated characteristics. To build the patterns, mobility was taken as the reference because it is the domain that most affected the population. The following groupings were made: a) mobility, psychological, and cognitive domains; b) mobility and psychological domains; c) mobility plus any other domain (excluding psychological); d) hearing plus any other domain (excluding mobility); and e) any domain with the exception of mobility or hearing.

**TABLE 1.** Intrinsic capacity screening tool adaptation of the available variables from the Mexican Health and Aging Study, Mexico, 2015

**Domain**	**Mexican Health and Aging Study question**
Cognition	Does not recall at least three words in the immediate word recall test Does not know the date (complete)
Psychological	Last week, the majority of the time: did you feel depressed? Last week, the majority of the time: did you feel that everything you did was an effort?
Hearing	Self-rated hearing, regular or poor (with or without hearing aid)
Vision	Self-rated vision, regular or poor (with or without glasses)
Vitality	Compared with two years ago, did you lose 5 kg or more? In the last two years, have you eaten less because of loss of appetite, digestive problems, or difficulties chewing or swallowing?
Mobility	Do you have difficulty walking several blocks? Do you have difficulty climbing several flights of stairs without resting?

***Source:*** Prepared by the authors based on the Mexican Health and Aging Study.

**Covariates.** The variables of sex, age, schooling (0 years, 1–6 years, and ≥7 years), and marital status (single, married/consensual union, separated, divorced, and widowed) were included as covariates in the socioeconomic field, along with current participation in the labor market and receipt of a pension. In the psychosocial field, the locus of control and satisfaction with life were analyzed. The eight locus of control items were added after reverse coding of the questions’ scores, further divided by 8, ending with an index that ranged from 1 to 4 (a higher score reflects greater internal locus of control and low score shows less control of one’s own life: 0–2 external, 3–4 internal) (18). The continuous scale of satisfaction with life (a high score meaning less satisfied with life) was included. Further, a social participation variable was constructed based on the person’s dedicating at least one hour to the following activities: caring for older people, caring for children, volunteering, training, attending sports, reading, playing mental games, playing board games, talking to other people, and craft-making. Categories were defined based on the number of activities carried out (0–1, 2–4, and ≥5 activities).

To control for the potential impact of health conditions on IC, the following chronic diseases were considered: hypertension and diabetes, individually; cancer, lung disease, heart attack, stroke, and arthritis into a variable with three categories (0, 1, and ≥2 chronic diseases). Also included were: self-rated health (divided into excellent/very good/good and regular/poor); frequent physical pain; falls (in the last two years); number of visits to a physician in the last year (0, 1–3, 4–11, and ≥12 visits); activities of daily living (walking, bathing, eating, going to bed, going to the toilet) were categorized into 0, 1, and ≥2 affected; and sleep problems (none, moderate, or severe).

**Statistical methods.** Descriptive statistics included an analysis of the differences between the sociodemographic characteristics and health conditions of the sample by age group and the affected domains stratified by sex and age. In order to establish association of socioeconomic, demographic, and health factors on IC impairment, two multinomial logistic regressions were modeled. The objective of the first model was to identify the factors associated with the number of affected domains, while the second model had the objective of analyzing the factors associated with specific profiles of affected domains (see the details in the section on IC screening, above). All analyses were done using the weights provided by MHAS in order to have a complete picture of the magnitude of this phenomenon.

## RESULTS

The sample of the 2015 round of MHAS consisted of 14 779 people, of which 12 459 (84.3%) had complete information for all IC domains. Analyzing the characteristics of the sample, generational differences stand out: people aged 80 and over (12.6% with more education) did not have the same access to education as the youngest (50.2% with more education) (Table 2). Likewise, as age increases, the percentage of people who work decreases, and that of people with a pension increases. Regarding the psychosocial field, external locus of control also increases with age, while social participation decreases. In relation to health conditions, as age increases, a greater negative self-perception of one’s health is observed, possibly associated with a higher burden of chronic disease, physical pain, functional dependence, and sleep problems.

Analyzing the number of affected domains, the results show that the Mexican population manifests possible decline in three main domains: mobility, visual, and psychological (Table 3). Regarding mobility, as age increases, greater deterioration occurs, and this is more evident among women. By sex, men have more vision problems between 50 and 79 years of age than women. In the psychological domain, there is a direct relationship with age, and the proportions affected are higher among women. In the cognitive domain, difficulties remembering and locating oneself in time increase with age, with 28% of adults aged 50–59 years having problems in the cognitive domain. In relation to hearing, a higher percentage of possible deterioration is found among males. Regarding vitality, it is clear that problems of loss of weight and appetite are less frequent overall (27.5%) but are of higher frequency among women. Analyzing the number of affected domains, the majority of the population aged 50 years or older had at least one domain affected (87.8%), reaching over 90% in the oldest group.

Analyzing the factors associated with the number of affected domains, which reflects the degree of decline of IC, no differences by sex were observed (Table 4). As age increases, the relative risk (RR) of having more affected domains increases significantly compared with not having any affected domain. Also, with longer length of schooling, the RR of having 4–6 affected domains decreases significantly by 17%. Regarding psychosocial factors, it is observed that perceiving less control over situations (external locus of control), greater sleep problems, greater dissatisfaction with life, and less social participation are related to a greater number of affected domains. Analyzing health conditions, self-perception of poor health, having a greater number of comorbidities, having physical pain, having had falls in recent years, and a greater number of visits to the doctor are related to a greater deterioration of IC. These results suggest that this proposal for an initial evaluation of possible deterioration of IC (in all its domains) relates to the deterioration of psychosocial and health conditions.

Analysis of the factors associated with the proposed profiles based on the observed patterns of at least two affected domains of IC reveals important findings (Table 5). Taking as the reference not having mobility and hearing affected, the RR of having at least some problem in the mobility, psychological, and cognitive domains (as the most unfavorable profile) increased significantly by 93% among women and by 7% as age increases, whereas the RR is reduced by 6% in those with more schooling. In addition, the RR of having this profile is associated with perceiving less control over what happens (22%), severe sleep problems (2.18 times), greater dissatisfaction with life (7%), and the chances of having greater social participation are reduced (38% for two or more activities). This profile is also associated with a worse self-perception of one’s health (2.44 times), with having hypertension (75%) and diabetes (22%), and of having two or more comorbidities (2.33 times), physical pain (2.93 times), and having suffered falls in recent years (37%). In general, this profile seems the most unfavorable, considering the health conditions affecting this population.

**TABLE 2.** Sociodemographic characteristics and health conditions of the included individuals, stratified by age group, Mexico, 2015

**Variable**	**Total**	**Age group (years)**			
		**50–59**	**60–69**	**70–79**	**≥80**
*N* (weighted)	17 704 457	7 106 788	5 994 048	3 374 396	1 229 225
Women	54.7	57.3	54.5	51.7	49.8
Years in school					
0	14.9	6.3	15.1	24.6	37.1
1–6	49.5	43.5	52.3	57.3	50.4
≥7	35.0	50.2	32.6	18.1	12.6
Marital status					
Single	4.9	5.2	5.0	3.8	5.3
Married/consensual union	67.7	73.5	71.4	59.1	40.2
Separated/divorced/widowed	27.4	21.3	23.6	37.1	54.5
External locus of control	33.9	28.0	34.7	40.5	47.5
Sleeping problems					
None	33.5	39.1	31.0	28.7	26.5
Moderate	53.2	49.6	55.7	55.3	55.4
Severe	13.3	11.3	13.3	16.0	18.1
Social engagement (number of activities)					
0–1	22.6	14.8	21.6	30.5	50.2
2–4	59.3	61.8	59.3	59.0	46.6
≥5	18.1	23.4	19.2	10.5	3.2
Regular/poor self-rated health	66.6	61.7	68.3	71.7	74.0
Hypertension	43.5	33.7	47.0	53.1	57.7
Diabetes	21.5	17.1	25.2	24.8	19.6
Number of other chronic diseases					
0	76.5	82.9	74.6	69.1	69.3
1	20.4	15.3	21.8	26.5	26.0
≥2	3.1	1.8	3.6	4.4	4.8
Frequent bodily pain	38.7	36.0	38.0	41.1	51.2
Falls in the last 2 years	42.7	39.3	42.1	47.6	51.8
Number of visits to a physician in the last year					
0	22.7	26.7	20.4	18.9	20.6
1–3	30.0	37.3	27.0	21.5	25.5
4–11	21.2	18.3	23.3	22.5	24.2
≥12	26.1	17.6	29.3	37.0	29.8
Affected activities of daily living					
0	87.6	92.9	88.7	81.5	68.5
1	7.3	5.0	7.2	9.6	15.1
≥2	5.1	2.2	4.1	8.9	16.4

***Note:*** The estimates were made from the sample that had complete information on the variables that constitute intrinsic capacity.

***Source:*** Prepared by the authors based on data from the Mexican Health and Aging Study.

In contrast to the above profile, still taking as reference people who have other domains affected except for mobility and hearing, the RR of having at least impaired mobility and having symptoms of depression (but without affected cognition), significantly increases in people with a higher education (4%) and in women (82%). Regarding the psychosocial domain, there are no differences in locus of control or in having greater participation in social activity. Regarding health conditions, the RR of having these affected domains does not increase significantly among those with diabetes; however, it is linked to having hypertension (73%), a greater number of comorbidities (3.47 times), physical pain, falls, and a greater number of visits to the doctor.

## DISCUSSION

To the best of our knowledge, this is the first thorough work on how the items included in the screening tool on IC from the WHO ICOPE guidelines associate with different features and how frequent they are. Notably, if the cut-off value suggested by these guidelines is used, almost 90% of the population will have to go into a second assessment. However, since WHO has a life-course focus, the cut-off value of having only one problem present could be useful in younger adults (e.g., individuals aged 50–60 years), where the prevalence of a positive screening is only 80%. It is noteworthy that mobility was the domain more frequently affected, with a clear relationship to age. Delving into those problems related to human movement and its decline with age has been a matter of interest in the field of aging (19). Moreover, different indexes aiming at exploring healthy aging have also reported an important impact in mobility (20).

**TABLE 3.** Frequency of individual affected domains and grouped, stratified by sex and age, Mexico, 2015

**Variable**	**Total**	**Total**				**Men**				**Women**			
		**50–59**	**60–69**	**70–79**	**≥80**	**50–59**	**60–69**	**70–79**	**≥80**	**50–59**	**60–69**	**70–79**	**≥80**
Mobility, *n* (%)	8 431 442 (47.6)	2 468 428 (34.7)	2 853 672 (47.6)	2 131 907 (63.2)	977 435 (79.5)	724 488 (23.9)	1 026 910 (37.6)	883 428 (54.3)	458 112 (74.3)	1 743 940 (42.9)	1 826 762 (56)	1 248 479 (71.5)	519 323 (84.8)
Visual, *n* (%)	7 931 130 (44.8)	2 949 413 (41.5)	2 488 581 (41.5)	1 767 139 (52.4)	725 997 (59.1)	1 288 009 (42.4)	1 201 113 (44)	888 494 (54.6)	352 509 (57.2)	1 661 404 (40.8)	1 287 468 (39.4)	878 645 (50.3)	373 488 (60.9)
Psychological, *n* (%)	7 627 029 (43.1)	2 705 841 (38.1)	2 502 136 (41.7)	1 729 348 (51.3)	689 704 (56.1)	939 088 (30.9)	890 766 (32.6)	761 001 (46.7)	282 882 (45.9)	1 766 753 (43.4)	1 611 370 (49.4)	968 347 (55.5)	406 822 (66.4)
Cognitive, *n* (%)	6 576 544 (37.2)	1 987 734 (28)	2 119 409 (35.4)	1 630 663 (48.3)	838 738 (68.2)	829 847 (27.3)	928 990 (34)	755 842 (46.4)	378 151 (61.3)	1 157 887 (28.5)	1 190 419 (36.5)	874 821 (50.1)	460 587 (75.2)
Hearing, *n* (%)	5 689 731 (32.1)	1 905 958 (26.8)	1 774 822 (29.6)	1 401 791 (41.5)	607 160 (49.4)	766 524 (25.2)	918 229 (33.6)	764 361 (46.9)	326 994 (53)	1 139 434 (28)	856 593 (26.3)	637 430 (36.5)	280 166 (45.7)
Vitality, *n* (%)	4 874 401 (27.5)	1 624 848 (22.9)	1 730 075	1 044 790 (31)	474 688 (38.6)	625 527 (20.6)	719 524 (26.4)	494 555 (30.4)	206 171 (33.4)	999 321 (24.6)	1 010 551 (31)	550 235 (31.5)	268 517 (43.8)
**Number of affected domains**													
0	2 165 769 (12.2)	1 282 164 (18)	684 254 (11.4)	182 724 (5.4)	16 627 (1.4)	674 732 (22.2)	350 773 (12.9)	109 975 (6.8)	15 137 (2.5)	607 432 (14.9)	333 481 (10.2)	72 749 (4.2)	1 490 (0.2)
1	3 836 990 (21.7)	1 804 219 (25.4)	1 404 108 (23.4)	508 054 (15.1)	120 609 (9.8)	793 158 (26.1)	769 689 (28.2)	270 272 (16.6)	60 595 (9.8)	1 011 061 (24.9)	634 419 (19.4)	237 782 (13.6)	60 014 (9.8)
2–3	7 416 673 (41.9)	2 823 592 (39.7)	2 645 703 (44.1)	1 490 412 (44.2)	456 966 (37.2)	1 202 116 (39.6)	1 104 917 (40.5)	708 000 (43.5)	282 213 (45.8)	1 621 476 (39.9)	1 540 786 (47.2)	782 412 (44.8)	174 753 (28.5)
4–6	4 285 025 (24.2)	1 196 813 (16.8)	1 259 983 (21)	1 193 206 (35.4)	635 023 (51.7)	367 924 (12.1)	505 144 (18.5)	540 335 (33.2)	258 804 (42)	828 889 (20.4)	754 839 (23.1)	652 871 (37.4)	376 219 (61.4)
**Profiles defined by affected domains**													
Mobility plus psychological and cognitive	2 368 324 (13.4)	490 245 (6.9)	685 845 (11.4)	704 776 (20.9)	487 458 (39.7)	146 475 (4.8)	194 864 (7.1)	261 002 (16)	188 965 (30.6)	343 770 (8.5)	490 981 (15)	443 774 (25.4)	298 493 (48.7)
Mobility and psychological	2 688 578 (15.2)	961 573 (13.5)	978 399 (16.3)	635 125 (18.8)	113 481 (9.2)	252 520 (8.3)	323 014 (11.8)	263 012 (16.2)	45 890 (7.4)	709 053 (17.4)	655 385 (20.1)	372 113 (21.3)	67 591 (11)
Mobility plus another not psychological	2 616 004 (14.8)	745 279 (10.5)	871 121 (14.5)	684 308 (20.3)	315 296 (25.7)	233 290 (7.7)	373 694 (13.7)	311 178 (19.1)	188 632 (30.6)	511 989 (12.6)	497 427 (15.2)	373 130 (21.4)	126 664 (20.7)
Hearing plus another not mobility	1 868 832 (10.6)	854 899 (12)	625 804 (10.4)	292 927 (8.7)	95 202 (7.7)	380 677 (12.5)	378 063 (13.9)	187 305 (11.5)	75 632 (12.3)	474 222 (11.7)	247 741 (7.6)	105 622 (6.1)	19 570 (3.2)
Any two not mobility nor hearing	2 159 960 (12.2)	968 409 (13.6)	744 517 (12.4)	366 482 (10.9)	80 552 (6.6)	557 078 (18.3)	340 426 (12.5)	225 838 (13.9)	41 898 (6.8)	411 331 (10.1)	404 091 (12.4)	140 644 (8.1)	38 654 (6.3)

***Source:*** Prepared by the authors based on data from the Mexican Health and Aging Study.

**TABLE 4.** Multinomial regression models for specific variables, with number of domains affected as the dependent variable and no affected domains as the reference group, Mexico, 2015

**Variable**	**1**		**2–3**		**4–6**	
	**RR**	**Sig.**	**RR**	**Sig.**	**RR**	**Sig.**
Age	1.02	***	1.03	***	1.07	***
Women	0.93		0.95		0.97	
Years in school	0.94	***	0.89	***	0.83	***
Married/consensual union	1.09		1.01		0.91	
External locus of control	0.92		1.11		1.20	**
Sleeping problems						
None (ref).						
Moderate	1.09		1.43	***	1.77	***
Severe	2.02	***	3.11	***	5.72	***
Low satisfaction with life	1.03		1.12	***	1.23	***
More social engagement ≥2 activities	0.85		0.63	***	0.43	***
Regular/poor self-rated health	1.40	***	2.86	***	5.48	***
Hypertension	1.17	**	1.27	***	1.42	***
Diabetes	1.08		1.26	***	1.68	***
Number of other chronic diseases						
0 (ref.)						
1	1.17		1.52	***	1.84	***
≥2	0.84		2.09	***	2.79	***
Frequent bodily pain	1.26	***	1.00	***	2.99	***
Falls in the last 2 years	0.90		1.14	**	1.38	***
Number of visits to a physician in the last year						
0 (ref.)						
1–3	1.06		1.03		1.17	
4–11	1.21	**	1.2		1.39	***
≥12	1.08		1.15		1.34	***
≥2 affected activities of daily living	1.77	**	4.36	***	5.75	***

RR, relative risk; Sig., statistical significance; ** *p* < 0.01; *** *p* < 0.001

***Note:*** Log likelihood = –11 809.738; *p* < 0.001; R^2^ (16.9%); BIC = 24 235; AIC = 23 751.48

***Source:*** Prepared by the authors based on data from the Mexican Health and Aging Study.

Since identifiable clusters had specific and multifactorial associations, it could be speculated that further assessment of those older adults will benefit from the proposed second step of ICOPE, with a particular emphasis on mobility and potential interventions. It is worth noting that activities of daily living were the variables with the strongest associations; this might point to a possible sequence of events, with the potential to be detected in early stages to avoid accelerated decline.

Population aging is an unstoppable phenomenon that will continue challenging all aspects of human activities (21), in particular health systems. It is foreseeable that those systems with higher vulnerability in societies with abundant disparities will be the most affected (22). Therefore, having a strategic plan for the care of older adults that emphasizes the main role of geriatric assessment, and an iterative process of intervention and follow-up, will certainly increase the probability of a healthier life for older adults (23). According to our results, it appears that Mexican older adults have a high burden of those conditions included in the screening tool; this, in turn, could be used to estimate how this or other programs will have to be implemented. In addition, having this information will allow dissemination of knowledge on how Mexican older adults are aging.

As previously mentioned, preventive measures have the potential to enhance a successful aging. Implementing tailored interventions for those domains that seem to be affected could improve the quality of care of older adults. For example, whenever the vitality domain is affected, a thorough dietary plan along with physical activity could prevent having additional problems in other domains. This also should raise flags for those who take care of older adults, not only to implement these interventions but also to ensure follow-up.

We acknowledge that our study has a number of limitations that the reader needs to take into account to appropriately interpret our results. This is a secondary analysis, from a study not designed to assess IC, selecting those questions that more closely reflect the nature of IC and its domains. Our data could have the impact of memory bias, since information relies heavily on self-report. On the other hand, IC and ICOPE are emerging fields yet to be further explored, which led to decisions such as the arbitrary patterns chosen to group the domains, which no doubt require further examination. It is important to stress that our data were only on the Mexican population, and that it is well-known that aging is heterogeneous across different cultures. Nevertheless, this field is currently growing, with interventions already in place to be tested in the improvement of IC (24–26), showing the potential of our results to feed into new research. In addition, comparisons across other countries and on how IC is expressed are needed. Future research should also consider expanding the characterization of this construct whenever the available data allow. Finally, replication of our results on other populations will add consistency to the IC construct, which still has numerous gaps to be filled.

**TABLE 5.** Multinomial regression models for particular variables, with specific combination of domains affected as the dependent variable, with any two domains affected except mobility or hearing as the reference group, Mexico, 2015

**Variable**	**Mobility plus psychological and cognitive**		**Mobility and psychological**		**Mobility plus another not psychological**		**Hearing plus another not mobility**	
	**RR**	**Sig.**	**RR**	**Sig.**	**RR**	**Sig.**	**RR**	**Sig.**
Age	1.07	***	1.03	***	1.06	***	1.01	***
Women	1.93	***	1.82	***	1.78	***	0.76	***
Years in school	0.94	***	1.04	***	1.05	***	1.04	***
Married/consensual union	0.94		1.07		1.12		1.23	***
External locus of control	1.22	***	0.96		0.92		1.04	
Sleeping problems								
None (ref.)								
Moderate	1.48	***	1.67	***	1.03		1.03	
Severe	2.18	***	2.36	***	0.92		1.22	
Low satisfaction with life	1.07	***	1.08	***	0.96	***	1.00	
More social engagement ≥2 activities	0.62	***	0.98		0.91		0.93	
Regular/poor self-rated health	2.44	***	2.44	***	1.44	***	1.67	***
Hypertension	1.75	***	1.73	***	1.49	***	1.09	
Diabetes	1.22	***	1.73		1.05		0.87	
Number of other chronic diseases								
0 (ref.)								
1	1.47	***	1.70	***	1.49	***	1.04	
≥2	2.33	***	3.47	***	1.90	***	1.03	
Frequent physical pain	2.93	***	2.83	***	1.60	***	0.95	
Falls (at last 2 years)	1.37	***	1.20	***	0.98		1.11	
Number of visits to a physician in the last year								
0 (ref.)								
1–3	1.06		1.20		1.09		0.90	
4–11	1.23		1.48	***	1.25	**	1.02	
≥12	1.21		1.64	***	1.56	***	1.09	
≥2 affected activities of daily living	6.80	***	6.20	***	4.74	***	1.51	**

RR, relative risk; Sig., statistical significance; ** *p* < 0.01; *** *p* < 0.001

***Note:*** Log likelihood = –10 785.653; *p* < 0.001; R^2^ (13.5%); BIC = 22 360.2; AIC = 21 747.31

***Source:*** Prepared by the authors based on data from the Mexican Health and Aging Study.

Our results will certainly impact the design and implementation of policies oriented at improving primary care of older adults in Mexico and probably the wider region, since with this screening it is possible to detect the affected domains and propose specific priority care schemes. However, because of its novelty, there is still an information gap on this subject. Therefore, it is important to continue evaluating the ICOPE tool with other sources of information and in other contexts. On the other hand, our approach allowed clarification of potential associations with IC that merit further elucidation and depth. According to our study, any decrease in self-rated health or increase in the number of visits to a physician should raise flags and prompt intervention into tightening the control of chronic diseases and a thorough assessment of activities of daily living. This might halt the progression to an accelerated decline in IC and decrease the frequency of adverse outcomes. Finally, it is worth mentioning that there is great heterogeneity in the health conditions of the older population, which is associated with inequality in the region and may slow the implementation of these actions—mainly among those who are more vulnerable.

### Conclusion

Decreased levels of intrinsic capacity in Mexican older people are associated with less schooling, poor self-rated health, more chronic diseases, more visits to a physician, and problems with activities of daily living.

## Disclaimer.

Authors hold sole responsibility for the views expressed in the manuscript, which may not necessarily reflect the opinion or policy of the *RPSP/PAJPH* or those of the Pan American Health Organization.
